# From Construction Industry Waste to High-Performance Insulation: Sustainable Rigid Polyurethane Foams with Recycled Polyol

**DOI:** 10.3390/ma18174179

**Published:** 2025-09-05

**Authors:** Kinga Wieczorek, Łukasz Bobak, Przemysław Bukowski

**Affiliations:** 1 Institute of Agricultural Engineering, Wrocław University of Environmental and Life Sciences, 37 Chełmońskiego Str., 51-630 Wroclaw, Poland; przemyslaw.bukowski@upwr.edu.pl; 2 Selena Industrial Technologies Sp. z o.o., Pieszycka 3, 58-200 Dzierżoniów, Poland; 3 Department of Functional Food Products Development, Wrocław University of Environmental and Life Sciences, 37 Chełmońskiego Str., 51-630 Wroclaw, Poland; lukasz.bobak@upwr.edu.pl

**Keywords:** polyurethane, recycling, sustainability, glycolysis, rigid foam

## Abstract

This study investigates the feasibility of incorporating chemically recycled polyol (glycolysate), derived from semi-rigid polyurethane waste from the building industry, into rigid PUF formulations intended for thermal insulation applications. Glycolysis was performed using a diethylene glycol–glycerol mixture (4:1) at 185 °C in the presence of a dibutyltin dilaurate (DBTDL) catalyst. The resulting glycolysate was characterized by a hydroxyl number of 590 mg KOH/g. Foams containing 5–50% recycled polyol were prepared and described in terms of foaming kinetics, cellular structure, thermal conductivity, apparent density, mechanical performance, dimensional stability, flammability, and volatile organic compound (VOC) emissions. The incorporation of glycolysate accelerated the foaming process, with the gel time reduced from 44 s to 16 s in the sample containing 40% recycled polyol, enabling a reduction in catalyst content. The substitution of up to 40% virgin polyol with recycled polyol maintained a high closed-cell content (up to 87.7%), low thermal conductivity (λ_10_ = 26.3 mW/(m·K)), and dimensional stability below 1%. Additionally, compressive strength improvements of up to 30% were observed compared to the reference foam (294 kPa versus 208 kPa for the reference sample). Flammability testing confirmed compliance with the B2 classification (DIN 4102), while preliminary qualitative VOC screening indicated no formation of additional harmful volatile compounds in glycolysate-containing samples compared to the reference. The results demonstrate that glycolysate can be effectively utilized in high-performance insulation materials, contributing to improved resource efficiency and a reduced carbon footprint.

## 1. Introduction

Polyurethanes (PUs) are a versatile family of synthetic polymers widely used across numerous industries, most notably in the form of foams. The polyurethane foam market continues to grow steadily, with its global value estimated at USD 49.5 billion in 2023 and projected to reach USD 67.8 billion by 2028, reflecting a compound annual growth rate (CAGR) of 6.5% [1]. Polyurethane foams (PUFs) are classified into two main categories based on their mechanical properties and cellular structure: flexible (soft) foams with open-cell structures, and rigid (hard) foams with closed-cell structures. The latter are typically used as high-performance thermal insulation materials in the construction and refrigeration industries due to their excellent insulating and structural properties [2,3]. The increasing use of such foams is directly linked to the thermal modernization of existing buildings, which is considered the most effective long-term strategy for reducing energy costs. Moreover, well-insulated buildings contribute to improved indoor comfort and a reduction in greenhouse gas emissions, aligning with broader climate and sustainability goals [4]. The strategic importance of building insulation is further emphasized by numerous governmental and EU-level programs that offer financial incentives and subsidies for energy-efficient renovations. Initiatives such as Poland’s “Clean Air” program, along with various national and European funding schemes, are expected to drive significant growth in demand for high-performance insulation materials, including polyurethane foams, in the coming years [5,6]. These programs not only support the adoption of innovative insulation technologies but also make them more accessible to a wider group of property owners and developers. For this reason, it is essential to take a comprehensive approach both to managing the growing amount of PU waste and to promoting the use of materials with low volatile organic compound (VOC) emissions and reduced carbon footprints. When it comes to waste management, landfilling remains the most common disposal method, but it raises serious environmental concerns due to the long-lasting nature of PU, the risk of toxic emissions in case of fire, and the occupation of valuable land resources. Combustion, while reducing the volume of waste, contributes to greenhouse gas emissions and may release harmful byproducts. These challenges are further compounded by the thermoset nature of PU, which makes it more difficult to recycle than thermoplastics. The urgency to address PU waste is underscored by evolving legislative frameworks and sustainability initiatives. The European Union’s Circular Economy Action Plan and the European Green Deal aim to drastically reduce landfill use and promote recycling and resource efficiency in materials management [7,8]. Revised regulations, such as the modification of Directive 1999/31/EC, set ambitious targets for waste reduction and recycling rates, pushing industries to seek innovative solutions for PU waste valorization [9,10]. Therefore, chemical recycling remains the most promising development path, as it enables the recovery of basic raw materials that can be reused in the production of high-quality new materials [11]. The main chemical methods include hydrolysis [12,13], methanolysis [14], aminolysis [15], acidolysis [16,17], and glycolysis [18,19,20,21,22,23]. These processes offer the potential for closed-loop recycling and significant reductions in environmental impact. However, many of them are energy-intensive, require complex logistics and waste sorting, and face challenges related to scalability and cost-effectiveness. Among all chemical recycling methods, glycolysis is currently the most widely studied and applied. It involves breaking down polyurethane bonds using a short-chain glycol, typically in the presence of a suitable catalyst and elevated temperature [24]. The advantages of this method include relatively mild reaction conditions (typically 180–220 °C), low equipment and input requirements, and the availability of various low-cost catalysts. The most commonly used ones include amines, inorganic salts and hydroxides, inorganic acids, and phosphorus compounds [25].

This work aims to develop a sustainable rigid polyurethane foam based on recycled polyols obtained through the chemical recycling of polyurethane waste from the construction industry. This approach addresses both environmental and legislative imperatives by demonstrating the feasibility of circular solutions for rigid PU foams, supporting the reduction in landfill and incineration waste, and contributing to the decarbonization of the construction sector. The research focuses on evaluating the impact of the addition of glycolysate on the performance and mechanical properties of rigid polyurethane foam, as well as environmental aspects such as the emission of VOCs. The novelty of this study lies in the use of a recycled raw material obtained from semi-rigid polyurethane foam waste from the construction sector to produce a high-quality thermal insulation material that meets the standards required for building insulation applications (PN-EN 14315-1), making it suitable for spray application [26]. The proposed recovery process does not generate additional waste, as is often the case with processes that require extensive purification steps before the recycled polyol can be reused. Furthermore, the study is based on an alternative type of feedstock, filling a gap in the current literature. Another distinguishing aspect of this work is the focus on VOC emissions, an important yet often overlooked factor that significantly affects the commercial viability of polyurethane-based products in the building sector. It is also worth noting that the developed polyurethane foam was intended for spray application, which is rarely encountered in the context of recycled materials promoting a circular economy. Support for sustainable development, the principles of a circular economy, and the reduction in the carbon footprint of polyurethane-based products are key elements of current research and industrial efforts. Recycling polyurethane waste not only conserves resources and reduces dependence on fossil-based raw materials but also lowers greenhouse gas emissions and supports the transition toward a circular, resource-efficient economy. Therefore, any research focused on this direction represents a highly relevant and valuable topic that deserves further exploration.

## 2. Materials and Methods

### 2.1. Raw Materials

The waste foam subjected to the chemical recycling process was sourced from Selena Industrial Technology Sp. z o.o. (Nowa Ruda, Poland). This foam is intended for window and door mounting, gap filling, and insulation and is characterized by a low density of approximately 10–15 kg/m^3^. The waste materials used in this study are generated in post-production, including those from the Research and Development department. Diethylene glycol (DEG) and glycerol (GLY), used as the glycolysis agents, were obtained from P.P.H. Standard Sp. z o.o. (Lublin, Poland). The catalyst used, TIB KAT 218, which is chemically dibutyltin dilaurate (DBTDL), was sourced from TIB Chemicals AG (Mannheim, Germany). For the foam synthesis, in addition to the recycled polyol (RP), the following raw materials were used in component A: Rokopol^®^ RF551 (hydroxyl value: 420 mg KOH/g; viscosity: 4000 mPa·s) from PCC Rokita, blowing catalyst Dabco T (chemically N,N,N′-trimethylamino-N′-ethylethanolamine) from Evonik Industries AG (Essen, Germany), flame-retardant TCPP (chemically tris (chloropropyl) phosphate) from Cortex Chemicals Sp. z o.o. (Tarnów, Poland), silicone, which is responsible for the foam structure, Tegostab B84730 from Evonik Industries AG (Essen, Germany), and silicone, which improves the miscibility of the premix Rokafenol N8P7 from PCC Rokita (Brzeg Dolny, Poland). Water was used as the blowing agent. All recipe ingredients are presented in parts per hundred polyol (php) by weight. For component B, Desmodur 44V20L, which is polymeric 4,4′-diphenylmethane diisocyanate (pMDI) from Covestro AG (Leverkusen, Germany), with an isocyanate group content of 31%, was used. The isocyanate index was 110. The raw materials were securely sealed and kept for 7 days at standard laboratory conditions (T = 23 ± 1 °C; RH = 50 ± 5%) to minimize the effects of external variables on their characteristics.

### 2.2. Polyurethane Synthesis

Rigid polyurethane foams were synthesized using a two-step method. The samples were produced by first mixing component A (polyol, silicones, water, and catalyst) using a high-speed stirrer at 4000 rpm for a duration that allowed the premix to become homogeneous (30 s). Then, the appropriate amount of component B (pMDI) was added to the prepared blend, mixed for 3 s, and poured into a mold. Both components A and B were preheated to 45 °C to best simulate the application conditions of a high-pressure spray machine. This method allowed for free foam growth and enabled the measurement of basic parameters quickly and easily, without requiring large amounts of raw materials, as would be necessary when using a specialized spraying machine. The isocyanate (NCO) index for all PUFs was 110. The prepared samples were left to fully cure at room temperature for 24 h. After this time, the samples were cut for testing, always from the center of the foam, referred to as the core. The sample names were determined based on the material (PUF) and the amount of glycolysate used instead of the primary polyol. The PU foam formulations are presented in Table 1.

### 2.3. Characterization of PUFs

The apparent densities of the foams were calculated based on the mass and volume measurements of the samples, in accordance with the ISO 845 standard [27]. Characteristic process times, such as gel time, tack-free time, and rise time, were measured using a stopwatch directly during the foaming process according to ASTM D7487-24 [28]. The closed-cell content was determined by ISO 4590 [29]. The measurement was carried out using the gas pycnometry method on samples measuring 60 × 32 × 32 mm by calculating the percentage of closed cells based on the difference between the geometric volume and the volume accessible to gas. The thermal conductivity coefficient was measured at 10 °C and 0 °C (the hot plate was set to 20 °C and 10 °C, and the cold plate was set to 0 °C and −10 °C) following the EN 12667 standard [30]. The measurement was carried out using the LM.PLUS 305 device (StiroLab d.o.o., Ljubljana, Slovenia). The cellular structure was analyzed using a Keyence VHX-7000N digital microscope (Keyence Corporation, Osaka, Japan) equipped with a high-resolution lens at 150× magnification. The dimensional stability of the foam samples was tested in accordance with EN 1604 under two extreme temperatures: (−20 ± 3) °C and (70 ± 2) °C, with a relative humidity of (90 ± 5)% [31]. Initial dimensions of the samples were measured (100 × 100 × 100 mm^3^), after which they were exposed to the specified temperatures for 48 h. Once the samples returned to room temperature, their dimensions were measured again, and the percentage change in dimensions was calculated. The compressive strength was determined according to ISO 844 by compressing 50 × 50 × 50 mm^3^ samples in both parallel and perpendicular directions to the foam rise at 10% relative deformation [32]. The test was conducted using a Zwick Roell Z2.5 testing machine (ZwickRoell GmbH & Co. KG, Ulm, Germany) with a compressive force speed of 2 mm/s. The fire reaction of the samples was tested according to DIN 4201 [33]. The samples were exposed to a controlled flame applied at a 45-degree angle to the lower edge of the sample for 20 s in a standardized test setup. The combustion behavior, including ignitability and flame propagation, was observed. If the flame height does not reach 150 mm, the material can be classified as B2 (normal flammability), while if it exceeds 150 mm, the material is classified as B3 (highly flammable). The determination of the gross heat of combustion was carried out by ISO 1716 using laboratory equipment, including an IKA C200 calorimeter, an IKA C248 oxygen station, and a C5010 calorimetric bomb, all at a temperature range of 17–25 °C (IKA-Werke GmbH & Co. KG, Staufen, Germany), with a pressure of 30 bar [34]. The samples for testing were prepared in a consistent and controlled manner, ensuring the reliability and reproducibility of the results. Each measurement for the aforementioned tests was performed five times for each sample to ensure accuracy and precision. Analyses of VOC were performed using a GC–MS system (GC-MSGERSEL (GERSTEL, Mülheim an der Ruhr, Germany), Agilent 7890 coupled to a 5977 MSD (Agilent Technologies, Santa Clara, CA, USA)) equipped with a GERSTEL MPS autosampler and solid phase microextraction (SPME) module. From cured polyurethane foam, cylindrical plugs (~200 mg) were cored from the geometric center using a 5 mm diameter cork borer and placed into magnetic screw cap headspace vials (18 mm thread). Extraction was carried out using a Smart SPME Fiber DVB/C-WR/PDMS (Divinylbenzene/Carbon Wide Range/Polydimethylsiloxane) with a fiber thickness of 50/30 µm, fiber length of 10 mm, and dark gray color code. Separation was achieved on a DB-5ms capillary column (Agilent 122-5532; 30 m × 250 µm × 0.25 µm; −60 °C to 325/350 °C) with helium as the carrier gas in constant-flow mode (0.8406 mL/min; initial pressure: 4.99 psi; average linear velocity: 33.16 cm/s). The oven program was as follows: 35 °C (3 min); ramp at 5 °C/min to 150 °C (0 min); ramp at 10 °C/min to 300 °C (5 min); and maximum oven temperature of 350 °C. The inlet (Front SS Inlet He) was held at 280 °C in split mode (1:1; split flow) and the GC–MS transfer line was held at 250 °C. Volatile compounds were extracted with incubation at 60 °C for 60 min, fiber penetration was 40.00 mm, extraction time was 15 min, injection penetration was 44.00 mm, and desorption was 300 s. Fiber bakeout was applied with 1 min pre- and post-bakeout at 43.00 mm penetration. The mass spectrometer operated in full scan mode (50–550 *m*/*z*; threshold of 50–150 Hz), with no solvent delay. The ion source was at 230 °C, quadrupole was at 150 °C, electron energy was 70 eV, and EM voltage was 1132.94 V. Total run time was 46 min. The identification of compounds was performed by matching the obtained spectra to those in the NIST17 mass spectral library.

### 2.4. Chemical Recycling of Semi-Rigid PU Foams

Post-production waste of one-component semi-rigid polyurethane foam was shredded into pieces approximately 1 × 2 cm in size. As the glycolysis agent, a mixture of glycols was used: diethylene glycol and glycerol in a 4:1 weight ratio. The decision to combine these two glycols was made to increase the functionality of the resulting glycolysate, as high-functionality polyols are used in the synthesis of rigid PU foams to achieve the desired mechanical properties. In patent KR101061839B1, a glycolysis agent was chosen to include, in addition to a short diol, pentaerythritol or sorbitol to obtain a highly functional glycolysate [35]. The glycolysis process was carried out in the presence of a tin-based catalyst (DBTDL), at a concentration of 0.2 wt% relative to the PU waste. The reagents were mixed at a PU to glycol mixture weight ratio of 1:2. Table 2 presents a summary of the main parameters of the glycolysis process. The chemolysis process was initiated by charging the glycol mixture and catalyst into a 1 L round-bottom glass reactor equipped with a mechanical stirrer fitted with a semi-circular anchor blade measuring 120 × 80 mm, operating at 100 rpm, along with a thermocouple, a reflux condenser, and a heating jacket with manual temperature control. The entire process was conducted under a nitrogen atmosphere to ensure inert conditions. Once the reactor temperature reached 185 °C, the shredded PU foam was gradually fed into the reactor at an average rate of 4 g PU per minute. After the addition was completed, the reaction was continued under the same conditions for another 50 min, of which the first 20 min were still required for complete foam dissolution. These process parameters were selected as optimal based on preliminary tests, offering a balance between product quality and process economics. The resulting glycolysate was analyzed without further purification to reflect a realistic industrial recycling approach.

### 2.5. Analysis of Recycling Polyol

The hydroxyl number of the glycolysate was determined using a potentiometric titration method following the ASTM E1899-23 standard. The method involves the reaction of hydroxyl groups with an excess of toluene-4-sulfonyl isocyanate (TSI) in an aprotic solvent, followed by back-titration of the remaining isocyanate groups using a tetrabutylammonium hydroxide (TBAH) solution [36]. The acid number was determined according to ASTM D4662-20 using potentiometric titration with a standardized solution of potassium hydroxide (KOH) in isopropanol [37]. Viscosity was measured using a Brookfield CAP 2000+ cone/plate rheometer (AMETEK Brookfield, Middleboro, MA, USA) by EN ISO 3219-2:2021. The measurements were carried out at 25 °C using spindle S01 [38]. The water content was determined using Karl Fischer’s method, as specified in the PN-81/C04959 standard, with a TitroLine KF device (SI Analytics GmbH, Mainz, Germany) [39]. Elemental semi-quantitative analysis of the glycolysates was performed using inductively coupled plasma mass spectrometry (ICP-MS). The analysis was conducted to determine trace metal content. An ICP MS NexION 300D system (PerkinElmer, Waltham, MA, USA) was used for this analysis, which is equipped with a quadrupole mass spectrometer, capable of semi-quantitative elemental analysis. The recycled polyol, commercial polyol, and glycols were analyzed by FTIR spectroscopy using a PerkinElmer Spectrum Two spectrometer (PerkinElmer, Waltham, MA, USA) in the range of 650–4000 cm^−1^, with 20 scans collected for each spectrum.

## 3. Results and Discussion

### 3.1. Characteristics of Recycled Polyol

Table 3 presents the results of the hydroxyl number, acid number, and viscosity measured at 25 °C for the recycled polyol (RP) and for the commercially available polyol used as the main polyol component in rigid foam applications. As shown in the table, RP has a higher hydroxyl number due to the residual unreacted glycolysis agent (the hydroxyl number for DEG is 1058 mg KOH/g, and for glycerol, it is 1830 mg KOH/g). The acid number is significantly higher in the polyol derived from the glycolysis process, which is a natural occurrence since, in addition to the main transesterification reaction between the polyurethane and glycol, side reactions also take place, such as the glycolysis of urea groups, leading to the formation of carbamates and numerous aromatic amines [40].

**Figure 1:**
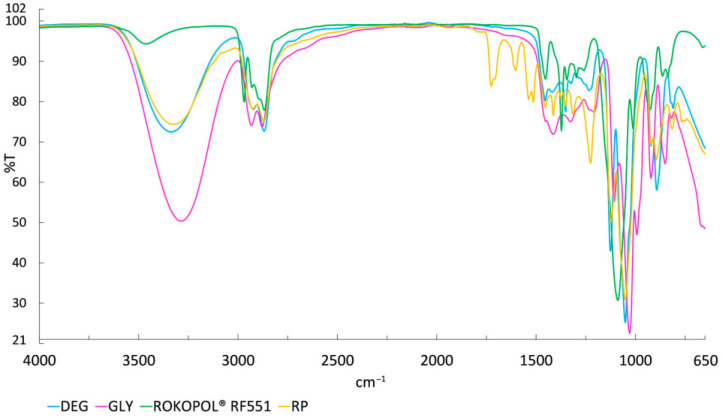
FTIR spectra of DEG, GLY, Rokopol^®^ RF551, and RP.

The transesterification reaction of urethane groups, which leads to the formation of new carbamates, proceeds faster than the reaction of urea groups, which results in carbamates and amines. These carbamates, in turn, undergo aminolysis due to amines generated during the glycolysis of urea groups, producing insoluble products. This process may lead to phase separation and the formation of two-phase glycolysates [41]. To confirm the origin of the high hydroxyl and acid values in the recycled polyol, the FTIR spectra of the RP sample, commercial polyol, and the glycolysis agents diethylene glycol and glycerol were analyzed. The spectra, presented in Figure 1, show characteristic differences in the intensity and distribution of functional bands. The most prominent of these, the broad O–H stretching band at 3325 cm^−1^ in the glycolysate, confirms a high concentration of hydroxyl groups, resulting from unreacted glycols [42]. The peaks at 2926 and 2871 cm^−1^ are attributed to the –CH stretching vibrations. The intense C–O–C stretching signals in the range of 1000–1200 cm^−1^, which were observed in all samples, are characteristic of polyether structures [43]. The band at 1725 cm^−1^ detected in RP corresponds to carbonyl groups (C=O) from urethane and urea bonds [20]. Residual aromatic structures originating from isocyanate are associated with the band at 1602 cm^−1^. The characteristic band at 1537 cm^−1^ is attributed to the coupling of symmetric deformation vibrations of bonded –N–H groups with the stretching vibrations of –C–N groups, as well as the deformation vibrations of N–H groups. The presence of urethane structures is evidenced by the N–H bending vibrations, corresponding to the band at 1513 cm^−1^ [44]. To thoroughly understand the chemical structure of the glycolysate, the most precise method is NMR spectroscopy. However, it is recommended only for purified glycolysate samples, as unpurified samples carry the risk of overlapping signals, making it difficult to unequivocally assign the peaks.

In order to reduce the acid number, which is undesirable in raw materials, the glycolysate would need to be purified. Common purification methods include extraction [17,45], distillation [46,47,48], or oxypropylation [25], a reaction with propylene oxide (PO) that leads to the formation of polyether polyols. Unfortunately, all of these methods are complex processes requiring specialized equipment and consequently involve significant financial investment. This approach does not align with the initial goals of the research, which aimed to develop a process suitable for large-scale implementation in the future.

The elemental screening analysis in the glycolysate obtained from polyurethane foam recycling revealed that its chemical profile, in terms of trace metals, differs dramatically from that of the commercially available polyol Rokopol^®^ RF551. As is well known, polyether polyols contain sodium and potassium impurities originating from their production process [49]. Apart from these elements, the commercial polyol is essentially free of other metals, with the remainder present at levels below 1 mg/L. In contrast, the glycolysate sample exhibited markedly higher concentrations, with the most abundant elements being phosphorus (300 mg/L), iron (140 mg/L), nickel (150 mg/L), bromine (100 mg/L), tin (116 mg/L), indium (100 mg/L), and chromium (200 mg/L). The presence of such elements is undesirable in the synthesis of polyurethane materials, as metal contamination in polyols can interfere with process control, reduce the quality of the final polyurethane, and may lead to unwanted side reactions or the excessive acceleration of the main reaction (primarily due to Fe, Cu, Zn, and Ni) [12,46,50]. For these reasons, the industry standard is to use polyols with a high degree of purity, whose trace metal content does not typically exceed 1–10 ppm, depending on the application requirements.

**Figure 2:**
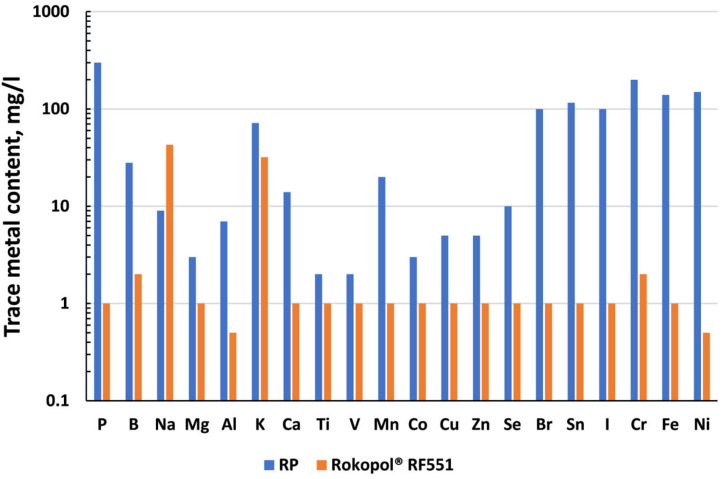
Comparison of the metal content in the recycled polyol sample and the commercially available Rokopol^®^ RF551.

Figure 2 presents a comparison of concentrations of individual metals found in the recycled polyol and reference polyol at levels exceeding 1 mg/L. The remaining elements occurred at concentrations below 1 mg/L. Although the glycolysate contains elevated levels of several elements, it may still be suitable for use in two-component polyurethane foam systems, as these are typically fast-curing formulations employing large amounts of highly reactive catalysts. Notably, the analysis did not detect above-average amounts of toxic heavy metals such as Pb, Cd, or Hg, which are regulated under REACH and RoHS regulations. Only the chromium content reached 200 mg/L; however, according to the REACH (EC 1907/2006) regulation, this remains within the permissible limit of below 0.1% in the final product [51].

### 3.2. Characterization of PU Foams Based on Recycled Polyol

#### 3.2.1. The Effect of RP on the Foaming Process of PUFs

The foaming process parameters of polyurethane foams containing recycled polyol were evaluated by measuring characteristic processing times: the cream time, gel time, tack-free time, and rise time (Table 4). Since glycolysate contains higher levels of metals than commercially used polyols, as well as amines originating from the breakdown of polyurethane bonds, it was expected that the secondary polyol would significantly accelerate the foam formation process [40]. Therefore, the recycled polyol was tested in formulations without an additional gelling catalyst, using only a blowing-type catalyst. To maintain the consistency of the results, the reference foam also contained only a single catalyst, which allowed for the evaluation of the real differences in the foaming process times and for the preparation of samples in the laboratory without the use of a specialized spray machine. Spray foams should be characterized by short start times to enable their easy application and uniform surface coverage. If the reference foam were to be a spray formulation, its recipe would have to include a gelling catalyst to accelerate the system. As expected, the addition of glycolysate accelerated the foaming process. At a glycolysate content of 40%, the gel time was almost 40% shorter than that of PUF_REF (with a gel time of 16 s). The results confirmed that when using recycled polyol, it is possible to avoid the use of one of the catalysts or significantly reduce its amount, which has a positive impact on the cost-effectiveness of the formulation, as catalysts are among the most expensive components of the recipe. In studies conducted by Amundarain and colleagues, it was demonstrated that with an increasing proportion of recycled polyols, the characteristic processing times became shorter, as was also observed in the present work [46]. It is also worth noting that high amounts of glycolysate slowed down the foaming process. This effect can be attributed to the high acid value of the recycled polyol (17.2 mg KOH/g), which may contribute to inhibiting the activity of the added catalyst. It is well established that the reaction between carboxyl groups (COOH) and isocyanate groups (NCO) proceeds considerably more slowly than the reaction between hydroxyl groups (OH) and isocyanate groups [52]. Studies conducted by Gotkiewicz and co-workers also demonstrated that the addition of recycled polyol gradually slowed down the reaction. This effect was attributed to the lower functionality of the polyol and its higher acidity [53].

#### 3.2.2. The Influence of Glycolysate on the Closed-Cell Content, Thermal Conductivity Coefficient, Apparent Density, and Cellular Structure of PUFs

The closed-cell content, thermal conductivity coefficient (λ), and apparent density were determined for polyurethane foams containing glycolysate and for the reference formulation (Table 5). The closed-cell content of all samples ranged from 80.0% to 91.5%, with the highest value observed for PUF_20%, which contained 20% recycled polyol (RP). It was noted that the addition of glycolysate up to 30% increased the closed-cell content. Only at 50% RP did this parameter decrease to 80.1%, which is still within the typical range for spray-applied thermal insulation foams. The thermal conductivity measured at 10 °C (λ_10_) ranged from 22.84 to 27.00 mW/(m·K), whereas at 0 °C (λ_0_), the values were slightly lower, between 21.72 and 25.39 mW/(m·K), reflecting the expected temperature dependence resulting from the reduced thermal motion of the gas. These results also correlate with the closed-cell content; higher closed-cell fractions were associated with lower thermal conductivity values.

The apparent density ranged from 40.0 kg/m^3^ (PUF_20%) to 48.7 kg/m^3^ (PUF_50%). At higher glycolysate contents, a slight increase in foam density was observed. A rise in apparent density results in a higher thermal conductivity coefficient, as the denser polymer matrix facilitates greater heat transfer through the solid phase [54]. The higher thermal conductivity observed in foams containing glycolysate does not disqualify these materials, as they still function effectively as thermal insulators. Moreover, this phenomenon is widely reported in the literature. For example, in their study, Gotkiewicz et al. observed that a 2.2% decrease in closed-cell content resulted in an increase in thermal conductivity of 2.5 mW/(m·K) [53].

**Figure 3:**
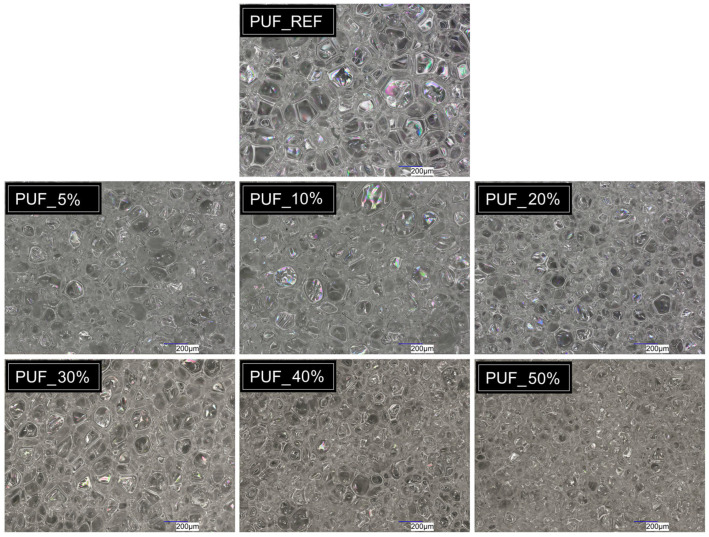
Photographs of the prepared PUFs from an optical microscope in the perpendicular direction to the foam rise. The scale bar is 200 μm.

Figure 3 shows optical microscope images of the foams taken with a high-resolution lens at 150× magnification. With increasing glycolysate content, the foams acquire a more beige and darker color, which is attributed to the dark coloration of the recycled polyol, containing a higher proportion of aromatic carbamate structures. Each sample presents a polyhedral configuration with minimized surface area, in accordance with Plateau’s description from 1873. Within these foams, slender bubble lamellae meet in symmetric clusters of three, at 120°, creating the so-called Plateau borders. The majority of the liquid concentrates at these borders and the foam vertices, whereas the films separating individual bubbles are regarded as extremely thin. This specific structural organization is essential for the stability and mechanical behavior of stiff foams [55]. In samples with an RP content above 30%, a thickening of the cell walls can be observed, which is likely related to the higher density of these foams. The average cell size was 172.44 μm for the reference foam and 116.77, 130.38, 112.67, 134.63, 116.15, and 99.19 μm for foams containing 5%, 10%, 20%, 30%, 40%, and 50% RP, respectively. The smallest cells were observed in the foam with the highest glycolysate content, which may be due to the good compatibility of the recycled polyol with the premix components or to the use of an additional silicone surfactant that facilitates phase mixing by reducing surface tension in component A [56]. It is possible that for this reason, the cells exhibited slightly smaller dimensions than those most commonly reported in the literature for foams with typical glycolysate additions (220–282 μm). This effect may be attributed not only to the formulation composition but also to specific foaming conditions, such as the mixing method, type of catalyst, surfactant selection, and system rheology. Among the factors mentioned above, it is believed that the most important was the use of the specific silicone Rokafenol N8P7 (a nonionic surfactant from the group of alkoxylated nonylphenols). Surfactants reduce the surface tension at the interface between gas and liquid phases, which helps stabilize the thin liquid films separating the foam bubbles. Selecting the right surfactant type and concentration, especially one exceeding the critical micelle concentration (CMC), enables the creation of foams that are more stable and characterized by smaller, finer bubbles [55,57]. Additionally, it is worth noting that in the conducted studies, the feedstock for the glycolysis process was a single-component semi-rigid PU foam, which has not previously been the subject of consideration by other researchers. The conducted studies indicate that the smaller cell size in samples containing recycled polyol had little impact on other foam properties. Regarding thermal conductivity, a decrease in the heat transfer coefficient was observed in samples with 5 to 30% recycled polyol content. However, in samples with a higher recycled polyol content, the values slightly increased. It is worth noting that this parameter also depends on density, which increased in those samples, indicating the consistency of the obtained results. Nevertheless, the observed trend is consistent with findings reported by other authors. Amundarain and co-workers confirmed that the incorporation of 15% recycled raw material reduced the cell size from 219–547 μm in the reference sample to 118–362 μm [46]. Similarly, in a study published in ACS Sustainable Chemistry & Engineering, rigid PU foams were produced with up to 50% substitution of commercial polyol by glycolyzed polyols. The resulting foams, with a density of approximately 40 kg/m^3^, exhibited cell diameters (depending on the growth direction) in the range of 220–430 μm. For foams containing 50% recycled polyol, the most frequent cell sizes were 222–260 μm, compared to 387–430 μm for the reference sample [58].

#### 3.2.3. The Influence of Glycolysate on the Compressive Strength and Dimensional Stability of PUFs

**Figure 4:**
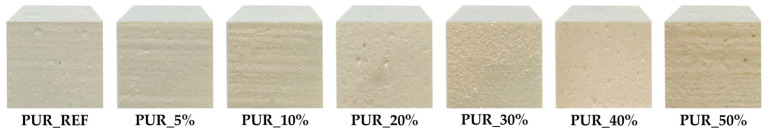
PU foam specimens with dimensions of 5 × 5 × 5 cm prepared for compressive strength testing. From left to right: reference sample and samples containing 5%, 10%, 20%, 30%, 40%, and 50% RP.

The mechanical properties of the prepared foams were evaluated by determining their compressive strength. The results for the compressive strength at 10% strain are presented in Table 6. Comparing the results for all samples, it can be observed that compressive strength values in the parallel direction are significantly higher than those in the perpendicular direction, which is a normal phenomenon resulting from the anisotropic cell structure of polyurethane foams. At low glycolysate contents (5–20%), a slight decrease in compressive strength was recorded, an effect commonly reported when incorporating recycled polyols. In the range of 40–50% glycolysate, a pronounced increase in compressive strength was observed, reaching 294 kPa and 262 kPa in the parallel direction. A similar increase was also observed in the perpendicular direction. A high proportion of glycolysate increases the viscosity of the system and results in finer foam cells, which in turn increases the apparent density of the foams and leads to a higher degree of crosslinking. Moreover, glycolysate contains a considerable amount of rigid structures, which can enhance its mechanical strength, provided that the formulation and catalyst system are optimized. Furthermore, the potential reaction of water with isocyanates could lead to the formation of rigid and durable structures (such as ureas and biurets), thereby increasing the crosslinking density and enhancing the compressive strength [59]. Figure 4 shows the samples of the prepared foams before the compression strength test. Donadini et al. demonstrated that replacing up to 30% of commercial polyol with glycolysate did not reduce the compressive strength compared to that of the reference sample but actually increased it. The authors reported an improvement of 20–25% relative to the foam without glycolysate, while maintaining cell isotropy and favorable thermal properties [20].

The dimensional stability of the rigid PUR foams was evaluated by measuring changes in their linear dimensions perpendicular to the foam rise direction (A1, A2) and parallel to the foam rise direction (A3), after conditioning at −20 °C and 70 °C for 48 h (Table 7 and Table 8). Changes in the dimensions of the samples after conditioning at different temperatures for 48 h were less than 1%, regardless of the measurement direction. Dimensional expansion was more pronounced at 70 °C, reaching 0.69% for the reference sample, whereas at −20 °C, the maximum change was 0.50% for the reference sample. This is likely due to the high relative humidity of 90% at 70 °C. The foams containing glycolysate exhibited better dimensional stability than the reference foam. This improvement can be attributed to the fact that the secondary raw material increases the foam density and results in smaller cells within the polyurethane matrix, leading to a higher degree of crosslinking. Furthermore, by definition, rigid foams exhibit better dimensional stability than flexible foams of lower density. For example, a commercial spray-applied PUR from SOPREMA company (Wadsworth, OH, USA) with a density of 40–50 kg/m^3^ exhibits dimensional changes ranging from −0.7% to +1.4% when tested at −20 °C, whereas at 70 °C and an RH of 97%, the changes range from +0.4% to +2.8% for high-performance systems and up to 7–13% for budget products [60]. In patent AU704932B2, rigid PUR foams were described as being produced from a mixture of virgin and recycled polyols (5–50 wt%). Dimensional stability was evaluated after 24 h of exposure at +70 °C (an RH of 95%) and −20 °C. The results demonstrated that foams containing glycolysate exhibited improved stability at low temperatures and at least comparable stability at high temperatures when compared to foams based on conventional polyols [61].

#### 3.2.4. Evaluation of Flammability of PUFs with RP

**Figure 5:**
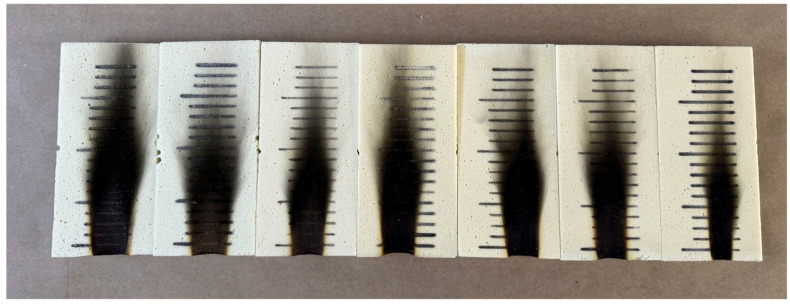
A comparison of samples after the flammability test conducted according to the DIN 4102 method. From left to right: PUF_REF, PUF_5%, PUF_10%, PUF_20%, PUF_30%, PUF_40%, and PUF_50%.

The flame height of rigid polyurethane foams was determined according to the DIN 4201 standard. The reference foam (0% recycled polyol) exhibited a flame height of 16 cm. The incorporation of glycolysate into the formulation led to a systematic reduction in the flame height: 14 cm (5% glycolysate), 13 cm (10% and 20%), 12 cm (30%), 11 cm (40%), and down to 10 cm for the 50% glycolysate foam. This clear downward trend indicates that foams containing recycled polyol obtained via glycolysis demonstrate improved fire resistance, as reflected by the slower flame spread. The enhanced performance observed for the glycolysate-containing foams can be attributed not only to changes in the chemical structure and potential char-promoting effects, but also to the chemical composition of the recycled component itself. The glycolysate used in this study was derived from waste rigid polyurethane foams that already contained flame retardants, including tris (2-chloropropyl) phosphate (TCCP), among others. It is therefore reasonable to assume that a portion of these flame retardants were transferred into the regenerated polyol and subsequently into the new foam structure, partially fulfilling the role of an additive flame retardant [62]. Polyurethane foams modified with phosphorus-based flame retardants exhibit a marked increase in fire resistance and a reduced tendency for flame propagation, which is associated with lower flame heights and decreased heat release rates [63]. Figure 5 presents one sample from each developed formulation after the flammability test. The results obtained for foams containing recycled polyol (RP) indicate, according to the relevant standard, that the foams meet the requirements for B2 classification. A B2 classification is achieved if the flame tip does not reach the reference line marked on the foam within 20 s (150 mm for bottom-edge application) [33]. This parameter is of particular importance to end users, as it directly relates to health and safety considerations, ensuring that the material limits flame spread and provides a higher level of fire protection in its intended applications.

The gross heat of combustion is another important parameter characterizing polyurethane foams due to its relevance to fire safety during the manufacturing, storage, and use of the final products. The obtained results for polyurethane foams containing glycolysate showed that the presence of this secondary raw material did not adversely affect the energetic properties of the material and, consequently, did not reduce its potential ignition resistance. The calorific values of all samples ranged from 26.3 to 28.4 MJ/kg, with an average of 27.05 ± 1 MJ/kg, which is typical for this class of materials. The highest heat of combustion was recorded for the reference sample (28.378 MJ/kg) and the sample with a 50% glycolysate content (28.238 MJ/kg), while the lowest values occurred for formulations containing 20–30% recycled polyol. Such fluctuations may be related to differences in the chemical structure of the polyurethane network arising from the presence of residues from chemical recycling, such as urethane products or low-molecular-weight polyol fragments. Nevertheless, even with a 50% glycolysate content, no significant increase in flammability was observed compared to the reference sample. These findings are consistent with literature data, in which Hasanzadeh et al. reported gross heat of combustion values of 33.18 MJ/kg for rigid PU foams and 32.45 MJ/kg for flexible PU foams [64]. Therefore, it can be concluded that the use of glycolysate as a partial replacement for polyol does not increase the fire hazard, which is crucial for its application as a thermal insulation material in construction or industry, in which flame resistance and user safety are of primary importance.

#### 3.2.5. The Influence of RP on VOC Release of PUFs

The preliminary testing of volatile organic compounds in polyurethane foams is a crucial step in assessing their quality and safety when in use. VOCs can significantly influence the health, environmental, and functional properties of polyurethane materials, particularly in terms of toxicity, odor, and the potential emission of harmful substances into the surrounding environment. Standards such as DIN ISO 16000-3:2013-01 [65] provide guidelines for precise, standardized assessment of VOC emissions; however, their implementation is often costly and time-consuming. Therefore, in the present study, a preliminary screening of VOCs was carried out on polyurethane foam samples containing different levels of glycolysate. Both reference samples and glycolysate-modified samples were analyzed using gas chromatography coupled with mass spectrometry to identify the main volatile components. The analysis revealed the presence of compounds such as isocyanates, specifically methyl isocyanate (methane, isocyanato-; with a retention time of 2.28 min), as well as phosphorus-based flame retardants, e.g., tris (chloroisopropyl) phosphate (TCPP; 2-propanol, 1-chloro-, phosphate; with a retention time of 31.57 min), which are used to reduce the flammability of the material. The first identified compound, methyl isocyanate, exhibited a match factor exceeding 75%, while the second, tris (chloroisopropyl) phosphate, showed a match factor above 78% in every analyzed sample. According to NIST17 guidelines, the minimum threshold for confident identification is a match factor above 70%. Therefore, other detected signals with lower match factors and no correspondence to polyurethane materials were excluded from further interpretation in the specific analysis.

**Figure 6:**
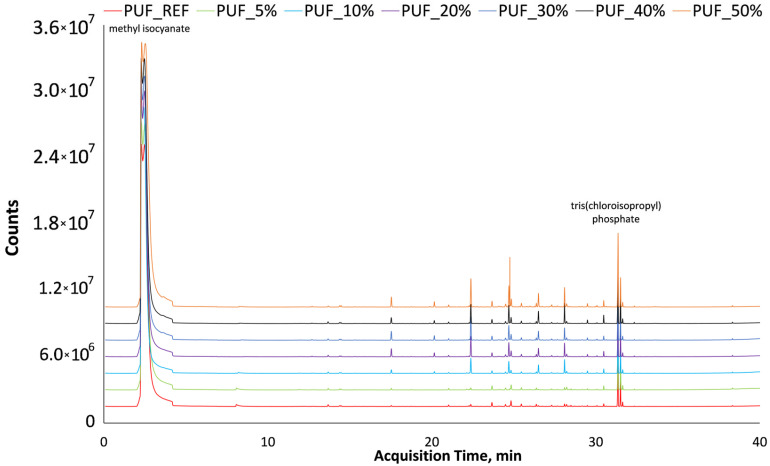
Comparison of GC-MS profiles for PUFs with RP and reference sample.

The presence of these two substances was consistently confirmed in all tested foam variants, including the reference sample. This finding indicates that the incorporation of recycled polyol does not promote the formation of new, undesired volatile compounds, nor does it worsen the emission profile compared to the base material. Figure 6 presents a comparison of the GC–MS profiles for all synthesized PUFs. The absence of additional, potentially harmful VOCs in the analyzed samples represents an important and positive indication in the preliminary assessment of glycolysate as an additive or secondary raw material for polyurethane foam production. While such screening tests do not replace full certification following international standards, they provide an efficient means of determining whether further, more comprehensive testing of glycolysate-containing materials is warranted. However, given the inherent limitations of qualitative screening, further quantitative VOC analysis is required to confirm emission levels and evaluate compliance with environmental certification schemes such as A+, AgBB, M1^®^, EMICODE^®^, LEED, or BREEAM.

Volatile compounds have long been the subject of investigation by numerous research groups. Parsons et al. analyzed polyurethane foams originating from the automotive sector using solid-phase microextraction combined with gas chromatography–mass spectrometry. Their study revealed that such foams contain a wide range of volatile compounds, including antioxidants, food preservatives, pesticides, plasticizers, and flame retardants. Among the detected plasticizers were tris (2-chloroethyl) phosphate and tris (3-chloropropyl) phosphate, compounds belonging to the same group as those identified in the foams examined in the present work [66].

## 4. Conclusions

This study demonstrated that chemically recycled polyol (glycolysate), derived from semi-rigid PU foam waste from the construction sector, can be successfully incorporated into rigid polyurethane foam formulations without compromising the key performance parameters required for thermal insulation applications. The addition of glycolysate influenced foaming kinetics, cell structure, and mechanical performance in a manner consistent with trends reported in the literature. The partial replacement of commercial polyol with recycled polyol (up to 40%) accelerated the foaming process, offering economic benefits by allowing a reduction in the amount of catalyst used in the polyol premix. The use of RP positively affected the cell structure, resulting in smaller cell sizes, which in turn increased the apparent density and improved mechanical properties. The foam containing 40% recycled polyol exhibited a 30% increase in compressive strength compared to the reference sample (PUF_REF: 208 kPa vs. PUF_40%: 294 kPa). A substitution of up to 40% commercial polyol with recycled polyol maintained a high closed-cell content (87.7%), low thermal conductivity values (λ_10_ = 26.3 mW/(m·K) and λ_0_ = 25.15 mW/(m·K)), and dimensional stability within the limits specified for spray-applied insulation systems (below 1%). The observed improvement in foam properties at higher glycolysate contents is likely due to increased crosslinking density, finer cell morphology, and the presence of rigid aromatic structures in the recycled polyol. The synergistic effect of these parameters positively influenced the functional properties, such as the mechanical and thermal performance, which are of utmost importance to the end users of the products. Flammability testing confirmed that all foams, particularly those with the highest glycolysate content, met the requirements for B2 classification according to DIN 4102, ensuring a level of fire safety appropriate for building applications. Preliminary VOC screening indicated no formation of additional, potentially harmful volatile compounds as a result of RP incorporation, suggesting that its use does not negatively impact emission profiles. However, this conclusion remains preliminary and should be verified through quantitative VOC analysis.

Overall, the results support the feasibility of using chemically recycled polyol as a partial substitute for virgin polyol in rigid PUF formulations, offering both environmental benefits and material cost reduction potential, while maintaining compliance with technical and safety standards relevant to the construction industry. Such a solution aligns with current trends in sustainable construction, which emphasize the effective integration of recycled materials in modern application systems, promoting the circularity of resources and minimizing the amount of construction waste throughout the product lifecycle. Modern building practices increasingly recognize thermal insulation as the only effective way to significantly reduce energy bills and environmental impact. Therefore, there is a growing need for eco-friendly insulation materials that not only have a reduced carbon footprint but also maintain high quality and performance. Programs like the European Green Deal are driving a shift towards sustainable construction by incentivizing the use of innovative, low-impact materials, and the developed polyurethane foam with glycolysate perfectly fits within these initiatives. It is also worth noting that the positive results obtained in this study confirm the possibility of valorizing problematic low-density polyurethane foam waste originating from a construction material manufacturing company. The implementation of such a solution enables the company to transition to a circular economy model, reducing waste generation and raw material consumption. Future work should include long-term durability assessments and full-scale environmental certification to confirm these findings under real-life application conditions. Moreover, the obtained results indicate that foams containing recycled polyol have strong potential for use in spray-applied systems. Even at the stage of preliminary laboratory tests, in which no specialized spray equipment was employed, the foams exhibited favorable properties, meeting the requirements of PN-EN 14315-1. Since spray application ensures more efficient mixing, which can further enhance the performance characteristics of the product, it would be valuable in future research to test the most promising formulation under actual spray application conditions.

## Figures and Tables

**Table 1:** Formulation of rigid polyurethane foams based on varying contents of recycled polyol.

	PUF_REF	PUF_5%	PUF_10%	PUF_20%	PUF_30%	PUF_40%	PUF_50%
Rokopol^®^ RF551, php	100	95	90	80	70	60	50
Recycled polyol, php	0	5	10	20	30	40	50
Dabco T, php	4.3	4.3	4.3	4.3	4.3	4.3	4.3
TCPP, php	27	27	27	27	27	27	27
Tegostab B84730, php	2.9	2.9	2.9	2.9	2.9	2.9	2.9
Rokafenol N8P7, php	2.9	2.9	2.9	2.9	2.9	2.9	2.9
Water, php	2.3	2.3	2.3	2.3	2.3	2.3	2.3
Index NCO	110	110	110	110	110	110	110

php: per hundred parts of polyol.

**Table 2:** Parameters of the recycling process.

Recycled Polyol Code	Glycolysis Agent	Weight Ratio Glycolysis Agent: PU	Catalyst	Amount of Catalyst (wt%/PU)	Temperature of Reaction (°C)
RP	DEG:GLY (4:1)	2:1	DBTDL	0.2	185

**Table 3:** Comparison of the parameters of recycled polyol and commercially available market polyol.

Polyol Name	Ohv, mg KOH/g	Av, mg KOH/g	η (25 °C), mPa·s	%H_2_O, wt%
RP	590 ± 20	17.2 ± 0.2	1500 ± 100	0.07 ± 0.02
Rokopol^®^ RF551	420 ± 20	0.08 ± 0.02	4000 ± 200	0.08 ± 0.02

**Table 4:** Cream time, gel time, tack-free time, and rise time of PUF foaming processes.

Foam Symbol	Cream Time (s)	Gel Time (s)	Tack-Free Time (s)	Rise Time (s)
PUF_REF	12 ± 1	44 ± 2	58 ± 2	50 ± 2
PUF_5%	10 ± 1	38 ± 2	50 ± 2	39 ± 2
PUF_10%	9 ± 1	37 ± 2	48 ± 2	39 ± 2
PUF_20%	9 ± 1	30 ± 2	38 ± 1	31 ± 2
PUF_30%	9 ± 1	23 ± 1	31 ± 1	24 ± 1
PUF_40%	9 ± 1	16 ± 1	22 ± 1	21 ± 1
PUF_50%	8 ± 1	20 ± 1	32 ± 1	18 ± 1

**Table 5:** Content of closed cells, thermal conductivity coefficient, and apparent density of PUFs.

Foam Symbol	Content of Closed Cell, %	Thermal Conductivity Coefficient at 10 °C, mWm·K	Thermal Conductivity Coefficient at 0 °C, mWm·K	Apparent Density, kg/m^3^
PUF_REF	88.3 ± 0.73	25.62 ± 0.16	24.39 ± 0.30	44.0 ± 0.83
PUF_5%	90.6 ± 0.72	22.84 ± 0.19	21.72 ± 0.35	42.0 ± 0.75
PUF_10%	90.4 ± 0.91	23.8 ± 0.16	22.86 ± 0.23	41.1 ± 0.54
PUF_20%	91.4 ± 1.15	23.6 ± 0.23	22.54 ± 0.25	40.0 ± 0.97
PUF_30%	89.0 ± 0.60	25.4 ± 0.26	24.37 ± 0.28	41.2 ± 1.18
PUF_40%	87.7 ± 2.30	26.3 ± 0.29	25.15 ± 0.27	44.9 ± 0.78
PUF_50%	80.1 ± 4.16	27.0 ± 0.50	25.89 ± 0.43	48.7 ± 1.21

**Table 6:** Compressive strength at 10% deformation of PUFs.

Foam Symbol	Compressive Strength, Parallel, kPa	Compressive Strength, Perpendicular, kPa
PUF_REF	208 ± 12	156 ± 10
PUF_5%	176 ± 14	144 ± 8
PUF_10%	170 ± 12	140 ± 11
PUF_20%	175 ± 16	141 ± 9
PUF_30%	193 ± 15	154 ± 13
PUF_40%	294 ± 14	203 ± 11
PUF_50%	262 ± 13	195 ± 8

**Table 7:** Dimensional stability of PUFs at −20 °C.

Foam Symbol	A1, %	A2, %	A3, %
PUF_REF	0.05 ± 0.15	0.07 ± 0.10	0.50 ± 0.36
PUF_5%	0.12 ± 0.08	−0.09 ± 0.06	0.39 ± 0.28
PUF_10%	0.07 ± 0.16	−0.05 ± 0.08	0.24 ± 0.21
PUF_20%	0.08 ± 0.11	0.07 ± 0.11	0.15 ± 0.25
PUF_30%	0.11 ± 0.12	0.06 ± 0.08	0.26 ± 0.19
PUF_40%	0.02 ± 0.06	0.00 ± 0.06	0.12 ± 0.17
PUF_50%	0.04 ± 0.05	0.02 ± 0.05	0.18 ± 0.19

**Table 8:** Dimensional stability of PUFs at 70 °C.

Foam Symbol	A1, %	A2, %	A3, %
PUF_REF	0.58 ± 0.11	0.15 ± 0.11	0.69 ± 0.45
PUF_5%	0.67 ± 0.12	0.12 ± 0.09	0.59 ± 0.36
PUF_10%	0.48 ± 0.16	0.09 ± 0.04	0.63 ± 0.33
PUF_20%	0.37 ± 0.13	0.11 ± 0.12	0.48 ± 0.19
PUF_30%	0.23 ± 0.17	0.13 ± 0.12	0.50 ± 0.25
PUF_40%	0.19 ± 0.10	0.10 ± 0.09	0.38 ± 0.34
PUF_50%	0.18 ± 0.08	0.08 ± 0.07	0.28 ± 0.24

## Data Availability

The original contributions presented in this study are included in the article. Further inquiries can be directed to the corresponding author.
